# Aging, sex and cognitive Theory of Mind: a transcranial direct current stimulation study

**DOI:** 10.1038/s41598-019-54469-4

**Published:** 2019-12-02

**Authors:** Mauro Adenzato, Rosa Manenti, Elena Gobbi, Ivan Enrici, Danila Rusich, Maria Cotelli

**Affiliations:** 1 Department of Psychology, University of Turin, Turin, Italy; 2 Neuroscience Institute of Turin, Turin, Italy; 3 Neuropsychology Unit, IRCCS Istituto Centro San Giovanni di Dio Fatebenefratelli, Brescia, Italy; 4 Department of Philosophy and Educational Sciences, University of Turin, Turin, Italy; 5 Department of Human Science, LUMSA University, Roma, Italy

**Keywords:** Geriatrics, Translational research

## Abstract

Aging is accompanied by changes in cognitive abilities and a great interest is spreading among researchers about aging impact on social cognition skills, such as the Theory of Mind (ToM). Transcranial direct current stimulation (tDCS) has been used in social cognition studies founding evidence of sex-related different effects on cognitive ToM task in a young people sample. In this randomized, double-blind, sham-controlled study, we applied one active and one sham tDCS session on the medial prefrontal cortex (mPFC) during a cognitive ToM task, including both social (i.e., communicative) and nonsocial (i.e., private) intention attribution conditions, in sixty healthy aging individuals (30 males and 30 females). In half of the participants the anode was positioned over the mPFC, whereas in the other half the cathode was positioned over the mPFC. The results showed that: (i) anodal tDCS over the mPFC led to significant slower reaction times (vs. sham) for social intention attribution task only in female participants; (ii) No effects were found in both females and males during cathodal stimulation. We show for the first time sex-related differences in cognitive ToM abilities in healthy aging, extending previous findings concerning young participants.

## Introduction

Normal cognitive aging is characterized by nearly linear declines from early adulthood in perceptual speed measures, memory and reasoning abilities until about age 65, when the decline accelerates^1,2^. A question of great interest is which impact aging has on social cognition skills, in particular on Theory of Mind (ToM)^3^. ToM refers to the ability to explain and predict other people’s behaviors in terms of the underlying mental states, such as beliefs, intentions, or feelings^4^. ToM abilities have been proposed to be based on a distributed neural network, including the medial prefrontal cortex (mPFC), the temporo-parietal junctions (TPJs) and the precuneus^5–7^. In recent years different studies highlighted the importance of the mPFC in communication, in particular for the pragmatic comprehension of a speaker’s intended meaning, such as, communicative intention attribution^8–12^. In previous studies, we found that while posterior regions of the ToM network are sufficient for the attribution of private intention (i.e., intention operating outside social interaction), only the attribution of social intentions (such as communicative intentions) recruited both posterior and anterior regions, in particular the mPFC^13–16^.

A large part of the scientific literature is focused on the normal development of ToM abilities during childhood^17,18^ and on ToM impairments in neurodevelopmental disorders^19–21^, in patients with acquired lesions^22,23^ and in neurodegenerative diseases^24–28^. Despite many evidence converge in clearly  describing ToM abilities development and impairment in atypical population, conflicting results regarding ToM were found during healthy aging^29–32^. For example, using the Reading the Mind in the Eyes (RME^33^), a task assessing the affective component of the ToM (i.e., the ability to infer other people’s emotions and feelings), Yildirim *et al*.^34^ recently found no differences between young (aged 18–28 years) and older (aged 51–80 years) adults. A similar result has been found by Girardi *et al*.^35^ using two ToM tasks evaluating both the affective and the cognitive (i.e., the ability to infer other people’s beliefs and intentions) component of the ToM. Indeed, these authors proposed the Faux Pas task^36^ and the Judgments of Preference task^37^ to younger (aged 18–23 years) and older adults (aged 60–81 years) finding no differences in both tasks. On the contrary, El Hay *et al*.^38^ assessed affective and cognitive ToM using, respectively, the RME task and the False-belief task^39^ showing  significant differences in both ToM components comparing the performance of younger (mean age = 23.13) and older (mean age = 69.53) adults. Lastly, Bottiroli *et al*.^40^ using the Faux Pas task found mixed results showing that young adults (aged 19–27 years) outperform both young-old adults (aged 60–70 years) and old-old adults (aged 71–82) on the cognitive but not on the affective component of ToM.

Behavioral studies indicated that females tend to obtain better performance than males on emotion recognition^41^, social sensitivity^20^, empathy^21^ and emotional intelligence^42^ tasks. In particular, for what concerns ToM, Baron-Cohen *et al*.^33^, Rutherford *et al*.^43^, and Schiffer *et al*.^44^ found that females, on average, perform better than males at the RME, a result confirmed by a meta-analysis revealing a small but statistically significant female advantage in judging mental states represented by eye stimuli^45^. Furthermore, Rutherford *et al*.^43^ showed that females outperform males in a scenario-based task requiring the ability to explicitly mentalize the reasons why an actor responded in a specific way in a real-life everyday situation. Interestingly, these sex differences in ToM abilities seem to be supported by differences in brain activity, as Frank *et al*.^46^ found that females, on average, activate the mPFC more than males during false-belief reasoning, and Krach *et al*.^47^ found larger mPFC activation for females during a “Prisoner’s dilemma” task.

In recent years, transcranial direct current stimulation (tDCS), a safe and well-tolerated neuromodulation technique^48^, has been used to study social cognition ability^49–54^. Based on polarity (anodal or cathodal) and on the initial neural activation state of the stimulated regions, tDCS can increase or decrease cortical excitability, although polarity-specific effects are not clear-cut^55^ and cathodal stimulation often results in weaker effects^56,57^. Effects of tDCS polarity on cortical excitability primarily concerns the stimulation of motor cortex, although several factors can turn facilitatory changes into inhibitory, and viceversa^58–60^. Overall, tDCS effects depend on several physical parameters including: current density, stimulation duration, the orientation and focality of the active target field, its projection areas, the resting surrounding structures and individual genetic polymorphisms^61–63^.

TDCS studies have rarely explored sex differences in social cognition^64,65^. In a previous tDCS study we found evidence of sex-related different effects on cognitive ToM in a group of young participants. In particular, using a cognitive ToM measure, assessing the ability to represent other people’s intentions from the observation of their daily actions^49^, we identified a significant interaction between sex and tDCS condition, with improved performance during anodal tDCS over the mPFC in females only^49^. Accordingly, a recent study^53^ showed improved performance on the RME^33^ task after the application of high-definition tDCS to the dorsomedial prefrontal cortex in young females only.

The main aim of the present study is to investigate by means of tDCS possible sex-related differences in cognitive ToM abilities in healthy aging. For this reason, we conducted a double-blinded study, applying tDCS on the mPFC (anodal, cathodal and sham tDCS) to modulate elderly participants’ performance on a cognitive ToM task. As in our previous study^49^, we assessed the ability to represent other people’s intentions from the observation of their daily actions, requiring participants to demonstrate their comprehension of short videos choosing the appropriate story ending. On the basis of existing literature indicating (i) sex-related differences in ToM abilities, (ii) the pivotal role played by the mPFC in cognitive ToM, in particular in processing communicative intentions, we expected to find sex-related differences in the effects induced by anodal tDCS over the mPFC on ToM performance, specifically for communicative intention processing, also in our group of healthy elderly participants. Moreover, we expected a reduction or no effects on ToM abilities in the cathodal condition in agreement with previous literature^56,66^.

Between October 2017 and January 2019, sixty healthy older adults (30 females and 30 males) were enrolled in this randomized, double-blind, sham-controlled study. Participants were randomized in two groups:

- anodal vs. sham tDCS (15 females and 15 males): participants underwent one active tDCS session and one sham tDCS session with the anode over mPFC and the cathode positioned between Oz and Inion;
- cathodal vs. sham tDCS (15 females and 15 males): participants underwent one active tDCS session and one sham tDCS session with the cathode over mPFC and the anode positioned between Oz and Inion.

During each tDCS session, participants saw at the PC a video version of a cognitive ToM task. The tDCS group assigned to each participant was obtained by stratified randomization according to Mini Mental State Examination and age. All participants and the experimenter were blind to the type of tDCS applied.

## Results

Regarding demographic variables, neuropsychological and clinical scores, the four groups were different on Geriatric Depression Scale (GDS) score and on verbal long-term memory tests (Rey Auditory Verbal Learning Task, immediate and delayed recall). In particular, female obtained higher GDS scores (though within the normal range) and better memory performance than male individuals (GDS: Anodal vs. sham tDCS males vs. female: U = 84, z = −1.16, p = 0.25; Cathodal vs. sham tDCS males vs. female: U = 63, z = −2.01, p = 0.044; Rey Auditory Verbal Learning Task, immediate recall: Anodal vs. sham tDCS males vs. female: U = 85, z = −1.12, p = 0.26; Cathodal vs. sham tDCS males vs. female: U = 53, z = −2.43, p = 0.015; Rey Auditory Verbal Learning Task, delayed recall: Anodal vs. sham tDCS males vs. female: U = 74, z = −1.56, p = 0.12; Cathodal vs. sham tDCS males vs. female: U = 55, z = −2.35, p = 0.019). The four groups were similar on the other neuropsychological and clinical assessments (see Table 1). Regarding RME task^33^, the overall group reached a mean of 21.9 SD 4.2 points (range = 15–29) indicating age-adequate ToM abilities and no differences between groups emerged (Anodal vs. sham tDCS males vs. female: U = 84, z = −1.16, p = 0.25; Cathodal vs. sham tDCS males vs. female: U = 101, z = −0.44, p = 0.663). See Table 1 for details.

**Table 1:** Demographical, clinical and neuropsychological data of sample group.

	Anodal vs. sham tDCS (n = 30)		Cathodal vs. sham tDCS (n = 30)		p-value*	Cut-off
	Male (n = 15)	Female (n = 15)	Male (n = 15)	Female (n = 15)		
Age (years)	68.3 (5)	67.5 (7)	67.1 (4)	68.1 (5)	0.895	
Education (years)	10.4 (5)	11.6 (4)	11.1 (4)	11.4 (4)	0.882	
Interpersonal Reactivity Index (IRI), total score	88.5 (8)	90.0 (8)	87.1 (9)	91.9 (10)	0.569	
Reading the Mind in the Eyes Test (RMET)	21.1 (4)	23.1 (3)	20.7 (5)	21.4 (4)	0.534	
**Mood and Anxiety Assessment**						
Geriatric Depression Scale (GDS)	2.4 (2)	4.8 (5)	2.5 (2)	5.1 (4)	0.120	<11
State-Trait Anxiety Inventory (STAI)-State	30.4 (7)	31.9 (7)	30.1 (6)	31.3 (6)	0.600	
State-Trait Anxiety Inventory (STAI)-Trait	33.9 (7)	36.2 (9)	32.7 (6)	38.9 (10)	0.350	
**Subjective Memory Complaints**						
Everyday Memory Questionnaire (EMQ)	46.1 (15)	45.5 (15)	46.9 (11)	44.9 (11)	0.918	
**Cognitive Reserve**						
Cognitive Reserve Index (CRI), total score	111.3 (15)	116.4 (16)	110.5 (16)	117.1 (15)	0.553	
**Cognitive Assessment**						
**Screening for dementia**						
MMSE	28.9 (1)	28.9 (1)	28.5 (1)	29.1 (1)	0.618	≥24
**Non-Verbal Reasoning**						
Raven’s colored progressive matrices	30.8 (3)	29.7 (5)	30.4 (3)	30.9 (4)	0.896	>17.5
**Memory**						
Digit Span (forward)	5.6 (1)	6.0 (1)	5.3 (1)	5.9 (1)	0.489	>4.25
Story Recall	12.5 (3)	14.0 (4)	12.0 (4)	14.0 (3)	0.432	>7.5
RAVLT (Immediate recall)	44.0 (8)	47.6 (7)	44.3 (10)	53.2 (9)	**0.020**	>28.52
RAVLT (Delayed recall)	8.5 (2)	10.2 (3)	8.9 (2)	11.6 (3)	**0.028**	>4.68
Rey-Osterrieth Complex Figure (ROCF), recall	17.8 (6)	16.6 (6)	18.1 (6)	18.3 (3)	0.766	>9.46
**Language**						
Token Test	33.7 (2)	34.0 (1)	33.4 (2)	34.3 (1)	0.280	>26.25
Verbal Fluency, phonemic	36.0 (9)	39.1 (14)	35.4 (10)	42.4 (9)	0.291	>16
Verbal Fluency, semantic	49.1 (9)	48.9 (12)	50.1 (7)	48.1 (8)	0.959	>24
Naming Objects (B.A.D.A)	28.9 (1)	27.9 (2)	28.8 (1)	28.6 (1)	0.733	
Naming Actions (B.A.D.A)	26.5 (1)	25.5 (3)	25.9 (1)	26.4 (2)	0.471	
Wechsler Adult Intelligence Scale – Vocabulary	38.6 (13)	44.0 (7)	36.7 (13)	41.5 (8)	0.412	
**Praxis**						
Rey-Osterrieth Complex Figure (ROCF), copy	31.6 (3)	30.9 (4)	31.5 (2)	30.9 (3)	0.896	>28.87
**Attentional and Executive Functions**						
Trial Making Test-A (sec)	35.9 (7)	41.5 (15)	36.0 (8)	40.9 (15)	0.769	<94
Trial Making Test-B (sec)	137.2 (67)	118.8 (60)	149.1 (77)	114.7 (44)	0.204	<283
Stroop test – interference effect on time (sec)	24.5 (12)	23.9 (8)	25.5 (13)	24.0 (9)	0.904	<36.92
Stroop test – interference effect on errors	0.6 (1)	0.8 (1)	0.6 (1)	0.7 (1)	0.919	<4.24
Digit Span (backward)	4.6 (1)	4.7 (1)	4.3 (1)	4.5 (1)	0.558	>2.64
Wisconsin Card Sorting Test (WSCT) – Global score	66.5 (35)	58.5 (43)	77.5 (34)	48.5 (36)	0.250	<90.6
WSCT – Perseverative responses	21.4 (15)	22.2 (17)	26.3 (17)	19.8 (19)	0.647	<42.7
WSCT – Non Perseverative errors	20.9 (12)	17.1 (13)	24.1 (12)	14.1 (11)	0.095	<30.0
WSCT – Failure to maintain the set	1.5 (1)	1.0 (1)	1.5 (1)	0.9 (1)	0.207	<4
Flanker Task–Effect of Incongruency on RTs (ms)	174.4 (74)	134.1 (99)	174.2 (71)	137.4 (38)	0.366	
Flanker Task–Effect of Congruency on RTs (ms)	2.2 (54)	0.5 (45)	0.6 (56)	4.3 (42)	0.968	

Raw scores are reported (SD between parentheses). MMSE: Mini Mental State Examination, RAVLT: Rey Auditory Verbal Learning Test, B.A.D.A (Batteria per l’Analisi dei Deficit Afasici), sec: seconds, RTs: Reaction Times, ms: milliseconds.*p-value related to the Freedman Analysis comparing four experimental Groups (anodal vs. sham female participants group, anodal vs. sham male participants group, cathodal vs. sham female participants group and cathodal vs. sham male participants group). Values in bold indicate significant difference (p < 0.05). Cut-off scores according to Italian normative data are reported.

### Attribution of intentions task

Since group differences in GDS scores have been recorded, Attribution of Intentions (AI) task^49,67,68^ performance (accuracy and RTs) were analyzed using repeated-measures Analysis of Covariance (ANCOVA) which included two types of “stimulation” (active or sham, within participants), two types of “stimuli” (PInt and CInt, within participants) and four “Groups” (anodal vs. sham female participants group, anodal vs. sham male participants group, cathodal vs. sham female participants group and cathodal vs. sham male participants group, between participants) as factors and the GDS scores as covariate.

#### Accuracy analysis

No significant effect for “Group” (F_(3,55)_ = 0.71, p = 0.55, η^2^ = 0.04), type of “stimulation” (F_(1,55)_ = 0.30, p = 0.59, η^2^ = 0.01) and interactions between factors were found.

#### Reaction time analysis

**Figure 1:**
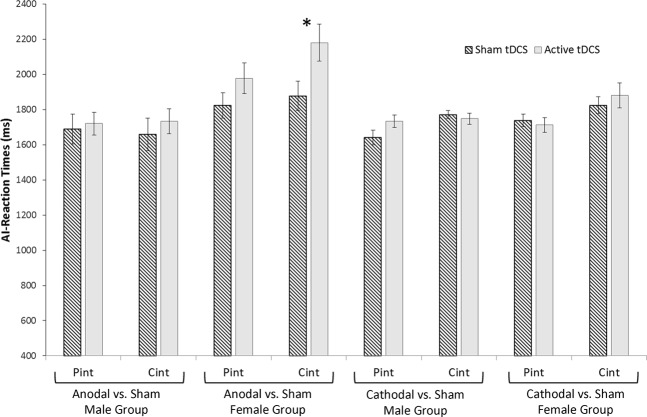
Changes in RTs in the AI task (Pint and CInt stories) for active and sham tDCS in the four experimental groups. Only in the female group that received anodal tDCS over mPFC the RTs during the CInt task were increased after active tDCS compared to sham stimulation. Asterisk indicates a significant effect (p < 0.05). Errors bars indicate mean standard errors.

RT analysis indicated a significant effect of type of “stimuli” (F_(1,55)_ = 5.69, p = 0. 021, η^2^ = 0.10), indicating shorter RTs for PInt than CInt stories (PInt = 1764.6 ms SE: 51.3; CInt = 1846.8 ms SE: 63.8). Moreover, the interaction between “Group”, types of “stimuli” and type of “stimulation” was significant (F_(3,55)_ = 2.93, p = 0.042, η^2^ = 0.13). No other significant factors or interactions between factors were recorded. Post-hoc analysis showed an increase of RTs during anodal tDCS as compared to sham tDCS selectively in the females group and selectively for CInt stories (PInt: 1978.1 ms SE: 118.5 [active tDCS] *vs*. 1823.5 ms SE: 99.3 [sham tDCS]; p = 0.773; CInt: 2180.8 ms SE: 146.7 [active tDCS] *vs*. 1878.5 ms SE: 122.4 [sham tDCS]; p < 0.001). No effects of cathodal tDCS were found for both sex samples group and no effects of anodal tDCS were recorded for the male (anodal vs. sham male participants group: PInt: 1732.3 ms SE: 118.5 [active tDCS] *vs*. 1706.9 ms SE: 99.3 [sham tDCS]; CInt: 1749.7 ms SE: 146.7 [active tDCS] *vs*. 1700.8 ms SE: 122.4 [sham tDCS], cathodal vs. sham female participants group: PInt: 1713.4 ms SE: 118.5 [active tDCS] *vs*. 1739.1 ms SE: 99.3 [sham tDCS]; CInt: 1881.6 ms SE: 146.7 [active tDCS] *vs*. 1825.2 ms SE: 122.4 [sham tDCS], cathodal vs. sham male participants group: PInt: 1761.2 ms SE: 118.5 [active tDCS] *vs*. 1662.1 ms SE: 99.3 [sham tDCS]; CInt: 1751.6 ms SE: 146.7 [active tDCS] *vs*. 1806.4 ms SE: 122.4 [sham tDCS]; all p-values > 0.74). See Fig. 1 for details. Interestingly, the four groups are similar in performance in Sham tDCS condition (all p > 0.30).

#### TDCS-sensations questionnaire

For each group, the tDCS sensations questionnaire scores reported during active tDCS were compared with those reported during the sham tDCS using a Wilcoxon matched pairs test showing comparable tDCS-induced sensations in the two stimulation conditions (anodal vs. sham female participants group = active: 1.58 SE 0.26, sham: 1.53 SE 0.3, T = 46.0, z = 0.80, p = 0.43; anodal vs. sham male participants group = active: 1.03 SE 0.3, sham: 1.20 SE 0.3, T = 51.5, z = 0.48, p = 0.63; cathodal vs. sham female participants group = active: 1.91 SD 0.3, sham: 1.47 SE 0.3, T = 31.0, z = 1.65, p = 0.10; cathodal vs. sham male participants group = active: 1.51 SE 0.3, sham: 1.20 SD 0.3, T = 33.0, z = 1.53, p = 0.13). Overall, only few subjects reported low intensity sensations (burning and itching).

## Discussion

The aim of the study was to investigate possible sex-related differences in cognitive ToM abilities in healthy aging. On the basis of the previous literature, we expected to find sex-related differences in the effects induced by anodal tDCS on the mPFC in the communicative intention component of the cognitive ToM task we used.

The findings of the present study showed that a single session of anodal tDCS over the mPFC of an aged female group led to significant slowing in RTs, compared to sham, in communicative intention processing, whereas cathodal stimulation induced no effects. No effects were found in males in both anodal and cathodal stimulation. Namely, female participants after anodal tDCS becomes slower to make decisions regarding the communicative intention component of the cognitive ToM task. However, the results revealed no significant effect of tDCS on cognitive ToM task accuracy. These findings suggest that tDCS may alter ToM processes, possibly making elderly female participants more uncertain about communicative intention attribution.

Interestingly, in the present study we found a significant slowdown in RT during the anodal tDCS over the mPFC in elderly female, while in a previous study^49^ we found a significant shortening of RT during the same kind of stimulation on the same brain area in young female.

We interpret our findings in light of the results recently presented by Emonson and colleagues^69^ using a single 20 min session of anodal tDCS to the prefrontal cortex in younger and older adults. To the best of our knowledge, this is the first and only study that used transcranial magnetic stimulation with electroencephalography to investigated local and global cortical reactivity changes following tDCS. One of the main findings of this study is a network level effects of the prefrontal tDCS in the posterior regions of the brain in younger adults (mean age = 24.50), but not in older adults (mean age = 65.47). According to the authors, these findings reflects higher propensity for a more dynamic response to the prefrontal tDCS in younger adults, with changes in the spread of electrical activity to distant regions. In the aging brain the prefrontal tDCS seems to significantly lose the capacity to modulate cortical reactivity in brain’s posterior regions^69^. This issue contributes to the interpretation of our findings about a selective effect of the tDCS applied over the mPFC on communicative (and not private) intentions processing in elderly participants, whereas the tDCS effect was observed on both private and communicative intentions in young participants involved in our previous work^49^. Given the results reported by Emonson and colleagues^69^ showing different effects of tDCS on cortical activity in younger and older healthy adults, our prediction of specific tDCS effect on communicative intention processing in elderly participants is reasonable as this kind of intention processing involves the mPFC, whereas the private intention processing involves exclusively the posterior areas^13,14^. Indeed, in a set of previous fMRI studies involving young individuals^13–15,70^, we demonstrated that an Intention Processing Network (IPN), including anterior region such as the mPFC, as well as posterior regions such as precuneus, and TPJs, are involved in comprehending intentions underlying action goals. More interestingly, we recently showed that the anterior region (the mPFC) is engaged in propagating the information to the other posterior regions of the network in a top-down mode, and receiving from these regions backward information in the context of a model of recirculation’s information^16^. Our results seem to corroborate the view that, in the aging brain, the top-down orchestration role of the mPFC significantly loses the capacity to modulate cortical reactivity in the brain’s posterior regions of the IPN.

We observed a significant RTs alteration in response to the ToM task in elderly females but not in elderly males. This result is in line with existing literature showing that advancing age is commonly associated with re-organization of fundamental brain networks, and the changes in both brain structure and function between younger and older adults are modulated by sex^71^. In particular, Zuo and colleagues^72^ demonstrated that higher-order cognitive regions exhibited decreased homotopic (i.e., the synchrony between geometrically corresponding interhemispheric regions) functional connectivity with age, and showed sex-related differences in the developmental trajectories of functional homotopy within dorsolateral prefrontal cortex, with a specific age-related decreases in functional connectivity for females only. Furthermore, Scheinost *et al*.^73^ explored sex differences in normal age trajectories of functional networks distributed across the brain and found that while both males and females show age-related decreases in functional connectivity in some networks, such as the default mode network, a divergent directions of aging trajectories characterize the fronto-parietal network with males showing increased connectivity with age and females showing decreased connectivity with age. Because these sex differences in normal brain aging may play a role in age-related changes in normal cognition, we suggest that this evidence contributes to explain why in the present study we found in elderly female, but not in elderly male, a significant slowdown in RT during the anodal tDCS over the mPFC.

Our data do not confirm the canonical assumption of anodal excitatory effects. This finding is in line with previous studies showing that anodal tDCS may exhibit differential effects during cognitive tasks^74–77^. In particular, recent researches reported increased RTs in a facial emotion identification task^75^ and greater difficulties in distinguishing between self and other faces^76^ induced by anodal tDCS. Moreover, we failed to find an inhibitory effect of cathodal tDCS applied over the mPFC. This finding is in agreement with a meta-analysis that found little evidence for an inhibitory effect of cathodal tDCS when applied during cognitive studies^56^. It has been suggested that tDCS effects might depend on the stimulated area^56^, type of the task^78,79^ and timing of stimulation^80,81^.

There are limitations of our study that need to be acknowledged. First, since our sample size was relatively small, findings reported here should be reproduced in larger cohorts. Second, as we did not vary the stimulation target, we cannot conclude for specificity of the mPFC-tDCS for the observed effects. Third, we did not use a non-mental control condition, such as for example a physical causality among objects. Lastly, we used a ToM task composed of two experimental conditions, one involving the mPFC (i.e., CInt) and one not involving this brain area (i.e., PInt) and we interpret our results in terms of two factors, that are age and sex. We cannot exclude that a different non-ToM task (e.g., executive functioning) engaging the mPFC in one condition but not in another could have given a similar pattern of results. Thus, future studies should clarify the domain-specific or the domain-general nature of the processes observed here.

In spite of these limitations, in the present work we show for the first time sex-related differences in cognitive ToM abilities in healthy aging, extending previous findings concerning young participants. Future brain stimulation studies in both clinical and healthy aging populations should take this finding into consideration when examining ToM and social cognition.

## Materials and Methods

### Participants

The sample size calculation was based on the tDCS effect (active vs. sham) induced in female group in our previous study on healthy young adults^49^. With a significance level (α) of 0.05, a power (1-β) of 0.9 (two-tailed independent t-test) and a correlation between assessments of 0.6, we obtained an effect size of 0.95 and, consequently, the minimum sample size was twelve participants for each group. Participants were excluded from the study if they had: (a) history of traumatic brain injury, brain tumors or stroke; (b) history of alcohol abuse; (c) prior or current neurological or major psychiatric disorders; (d) a pathological score in one or more of the neuropsychological tests; (e) hormone replacement therapy. Prior to their enrollment, participants were screened using a tDCS safety screening questionnaire and any contraindication to tDCS represented a further exclusion criteria. All participants underwent to a neuropsychological evaluation divided in two sessions, in order to verify the absence of any cognitive deficit before the tDCS sessions. See Table 1 for details.

The research was approved by the ethics committee of the IRCCS Istituto Centro San Giovanni di Dio Fatebenefratelli, Brescia and was conducted in accordance with the Declaration of Helsinki. Written informed consent was obtained from all subjects.

### Procedure

All participants performed two ToM tasks: the Reading the Mind in the Eyes (RME) task^33^ during the neuropsychological evaluation and the Attribution of Intentions (AI) task during active or sham tDCS. Moreover, all the participants underwent the RME task before the beginning of the two tDCS sessions, carried over by an assessor blinded to group allocation. The RME^33^ is a ToM task evaluating the subject’s ability to represent others’ mental states by observing eyes^33^. The participants were required to choose which word, out of four, better described the thinking or feeling of the character displayed in the photograph. The total number of correct choices (range: 0–36) is the RME task score. Participants were tested on RME before to the tDCS session to exclude participants with subtle ToM difficulties^82^.

### Attribution of intentions (AI) task

**Figure 2:**
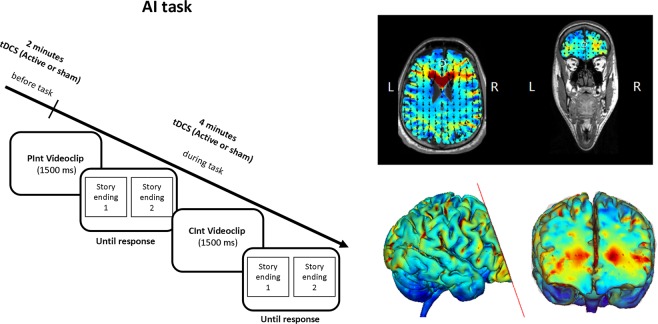
Experimental design and Current flow model for anodal tDCS application (anode over mPFC and cathode between Oz and Inion). Active or sham tDCS was started 2 minutes before the beginning of the experimental task and continued throughout the AI task. The anode was over the medial prefrontal cortex and the cathode placed between Inion and Oz. The device utilized two 7 × 5 cm sponge pads and the current flow model is represented in the transverse view and 3D view on the Male 1 model in Soterix HD Targets software (Soterix Medical). Arrows represent the direction of current flow. In the AI task, a short video was played, and the participant was asked to choose the picture representing a logical story ending by pushing one of the two buttons on the button box. One example for each stimulus condition (CInt and PInt) is displayed.

The AI task was used to test the effects of anodal and cathodal tDCS (vs. sham tDCS) on ToM abilities. The AI task is a previously used video version of a cognitive ToM task^49,67,68^. Participants were asked to choose the appropriate story ending by two picture (out of two concluding). The correct picture represented a probable conclusion, whereas the incorrect picture represented an improbable ending (see Fig. 2). The visual location of the correct answer was randomized and the two possible story endings were shown simultaneously until the participant responded. The items were displayed using Presentation software (Version 16.3, www.neurobs.com). Accuracy was recorded as the percentage of correct responses and the reaction time (RT) was recorded from beginning time of the two possible concluding pictures until the subject’s answer.

There were two experimental conditions: (a) the Communicative Intention condition (CInt), in which participants were required to recognize another person’s communicative intention during a social interaction; (b) the Private Intention condition (PInt), in which participants were required to recognize another person’s intention while watching his/her isolated actions. Each participant saw 34 video stories for each tDCS condition (68 stories in total) plus two additional training stimuli for each condition. The 34 CInt stories and the 34 PInt stories were split into two mixed blocks of 34 stimuli (17 PInt and 17 CInt stimuli) each corresponding to one of the two types of stimulation (active and sham stimulation). See Fig. 2. Each participant underwent one active and one sham tDCS session answering to the two corresponding blocks on 34 stories each.

The stimulation conditions (active or sham tDCS) and the order of the presentation of the two stimuli blocks were randomized across participants. The two tDCS sessions were administered on two consecutive days at the same time of the day.

### tDCS procedure

Active tDCS was applied using a battery-driven constant-current stimulator (BrainStim, EMS; Bologna, Italy) through a pair of saline-soaked sponge electrodes (7 cm × 5 cm). The target area for tDCS was the mPFC (Montreal National Institute coordinates: 0, 60, 18;^13–16,70^). For the healthy older individuals assigned to the anodal vs. sham tDCS group, during the active tDCS session the anode was placed over the mPFC (i.e., Fpz site) and the cathode was positioned between Oz and Inion, whereas in cathodal vs. sham tDCS group the active session involved a reversed montage with the cathode over the mPFC and the anode between Oz and Inion. See Fig. 2 for a graphical representation.

During active tDCS, a constant current of 1.5 mA was applied for 6 minutes (with a ramping period of 10 seconds at the beginning of the stimulation), starting 2 minutes before the beginning of AI task^49,67,68^ and covering all the task. The current density (0.043 mA/cm^2^) was maintained below the safety limits^83^. In the sham stimulation condition, the tDCS procedure was the same, but the current was turned off 10 seconds after the beginning of the stimulation and turned on for the last 10 seconds of the stimulation period, making this condition indistinguishable from the experimental stimulation. Active or sham tDCS were delivered after a numeric code was input into the device, allowing for blinding of the operator before and during the tDCS administration.

At the end of the stimulation session we asked to the participants to answer a questionnaire regarding the perceptual sensations they experienced during the active and sham tDCS sessions^84^ in order to test the blindness of the participants to the type of stimulation and to register potential side effects of tDCS.

### Statistical analyses

Statistical analyses were performed using Statistica software (version 10; www.statsoft.com). Considering that the data were not normally distributed, demographic variables, neuropsychological and clinical scores were compared between the four groups (anodal vs. sham female participants group, anodal vs. sham male participants group, cathodal vs. sham female participants group and cathodal vs. sham male participants group) using Friedman non-parametric statistical test and Mann-Whitney U Test.

AI task^49,67,68^ performance (accuracy and RTs) were analyzed using repeated-measures Analysis of Covariance (ANCOVA) which included two types of “stimulation” (active or sham, within participants), two types of “stimuli” (PInt and CInt, within participants) and four “Groups” (anodal vs. sham female participants group, anodal vs. sham male participants group, cathodal vs. sham female participants group and cathodal vs. sham male participants group, between participants) as factors and the GDS scores as covariate. Considering that the RTs data were not normally distributed (Kolmogorov-Smirnov Test: d = 0.10, p < 0.01; Skewness +1.5, right skewed), we adopted logarithmic transformation of RTs data. Post-hoc analysis was carried out using the Bonferroni correction for multiple comparisons.

The perception of sensations scores were compared between active and sham tDCS in each group using Wilcoxon matched pairs test.

Statistical significance was set at p < 0.05. Statistical power and effect size (Cohen’s d) analyses were performed using GPower 3.1^85^.

### Ethics statement

All procedures performed in studies involving human participants were in accordance with the ethical standards of the institutional research committee. Informed consent was obtained from all individual participants included in the study. Ethics approval was obtained from the local Ethical Committee (IRCCS Istituto Centro San Giovanni di Dio Fatebenefratelli, Brescia, Italy).

## Data Availability

All data and code are available upon reasonable request.
